# The Necessity of Diploid Genome Sequencing to Unravel the Genetic Component of Complex Phenotypes

**DOI:** 10.3389/fgene.2017.00148

**Published:** 2017-10-11

**Authors:** Fernando Aleman

**Affiliations:** Scripps Research Institute, La Jolla, CA, United States

**Keywords:** chromosome phasing, diploid alignment, diploid genomes, GWAS (genome-wide association study), structural variants, genetic variants, SNP association study, Diploid Manhattan Plot

Genome-Wide Association Studies (GWAS) correlate the genotype with the phenotype, identifying the genetic variants that are linked to any particular trait or disease. In 2005, a ground-breaking successful GWAS in humans associated the complement factor H gene with age-related macular degeneration (Klein et al., 2005). Since then, many successful GWAS using genotyping arrays have been published (Manolio, 2017), but due to the lowering cost of DNA sequencing, whole genome sequencing GWAS are becoming more frequent. However, the usefulness of classical GWAS has recently been questioned in a *Cell* publication (Boyle et al., 2017). The authors explain that genetic variants causing a disease should be part of a pathway connected with the etiology or prognosis of the disease, and moreover, they describe the benefits of linking GWAS with cell specific gene expression data. Still, many GWAS fail to correlate a specific genetic variant with a gene or a pathway leading to disease. This is partially due to the loose definition of how to establish an association between each genetic variant (frequently in non-coding regions) and the causal gene. In addition, the size of the effect of each genetic variant in polygenic traits and low penetrance genetic diseases is difficult to accurately establish due to confounding factors such as population stratification.

One of the main weaknesses of whole genome sequencing GWAS is the fact that for every diploid (or polyploid) organism we only obtain the “haploid genome.” Due to the prevalent short-reads technology, we merge both gene copies of every chromosome into one, losing physical connections and proximity between genetic variants in homologous chromosomes. Integrating both allele sequences as if they were one hampers the elucidation of haplotype specific structural variants (SVs). Indeed, SVs are more frequent in one haplotype vs. homozygous SVs (Sudmant et al., 2015; Hehir-Kwa et al., 2016). In addition, linkage disequilibrium and genetic linkage are difficult to accurately elucidate when the homologous chromosomes are merged, which decrease the power of many gene- and pathway-based association studies (Mooney et al., 2014).

To solve this issue, there have been several studies reporting the separation of alleles into chromosomes (phased chromosomes) of several genomes, but so far, only four studies have reported *de novo* human diploid genomes (Levy et al., 2007; Cao et al., 2015; Seo et al., 2016; Weisenfeld et al., 2017). In the paper *Direct determination of diploid genome sequences*, Weisenfeld et al. recently demonstrated that an accurate and cost effective method can be routinely used with the most popular Illumina sequencing technology. However, this method has only been tested on human genomes and some difficulties may arise for other species. In fact, it is worth to mention that GWAS have been widely used in plants (Korte and Farlow, 2013; Huang and Han, 2014) where the polyploidy of some species can introduce even more noise in the final haploid sequence. Thus, the benefits of using diploid (or polyploid) genomes materialize in two ways. First, better disease/trait variant calling (since we would have the real genome without noise coming from the “mix and match” of homologous chromosomes). Still, a high number of diploid genomes would increase the statistical power for the identification of new variants causing disease or a trait. The second advantage is the potential to detect protective genetic variants which, as mentioned below, are now potentially actionable with CRISPR/Cas9 in combination with correcting the faulty variant. Other general benefits can come from closing the “missing heritability gap” problem, and a better quantification of penetrance. So far, we do not fully understand the reasons for incomplete penetrance of most genetic diseases. Indeed, the analysis of diploid sequences has the potential to modify how we measure penetrance, since we would be able to include in the analysis not only the genetic variant that directly cause disease, but also any other protective variant that might co-exist in *cis*. Even concepts such as *conditional full penetrance* may arise (conditional to the sequence in a different interacting locus).

Diploid genomes are not only required for understanding allele-specific expression, but also to understand the real output of each allele. Two frameshifts in a gene will have completely different outcomes if they are both in the same allele or if each frameshift occurs in a separate allele. In addition, penetrance levels should be determined based not only on one genetic variant, but also on the genetic variants occurring in close proximity that are in linkage disequilibrium. This is particularly important for genetic diseases with incomplete penetrance such as celiac disease and allele-specific diseases such as Huntington's disease. Furthermore, with a high quality diploid sequence, CRISPR/Cas technology provides a potential actionability in two different ways. Allele-specific diseases can be precisely targeted, without affecting the healthy allele (Paquet et al., 2016), and diploid genomes may enable the discovery of allele-specific protective genetic variants, which could be targeted with CRISPR to improve health. Examples of how phasing loci improve the identification of disease causing variants are still limited but increasing (Safrany et al., 2013; Sharp et al., 2016; Subramanian et al., 2017). Plant breeding programs will also benefit from phased chromosomes since many important crops are polyploid and the genetic makers for heterosis may be revealed with polyploid sequences (Chen, 2013; Minio et al., 2017).

Although the cost of obtaining a diploid genome could cease to be a problem, other challenges lie ahead. Finding a proper reference for comparison will be daunting. However, the analysis may be split in two steps. First, each homologous chromosome of a diploid genome can be compared to a reference “haploid genome” obtaining a “Diploid Manhattan Plot” (Figure 1). The benefits of choosing a stratified population as reference need to be elucidated yet. Then, the selected loci in individual chromosomes with higher statistical significance should be explore in detail and compared with control diploid loci. When causes and controls are used, this method would work to reveal not only the causal genetic variants, but also potential protective variants from low penetrance diseases. Finally, comprehensive graphical models will be needed along with the human resources required to analyse, interpret and provide genetic counseling.

**Figure 1:**
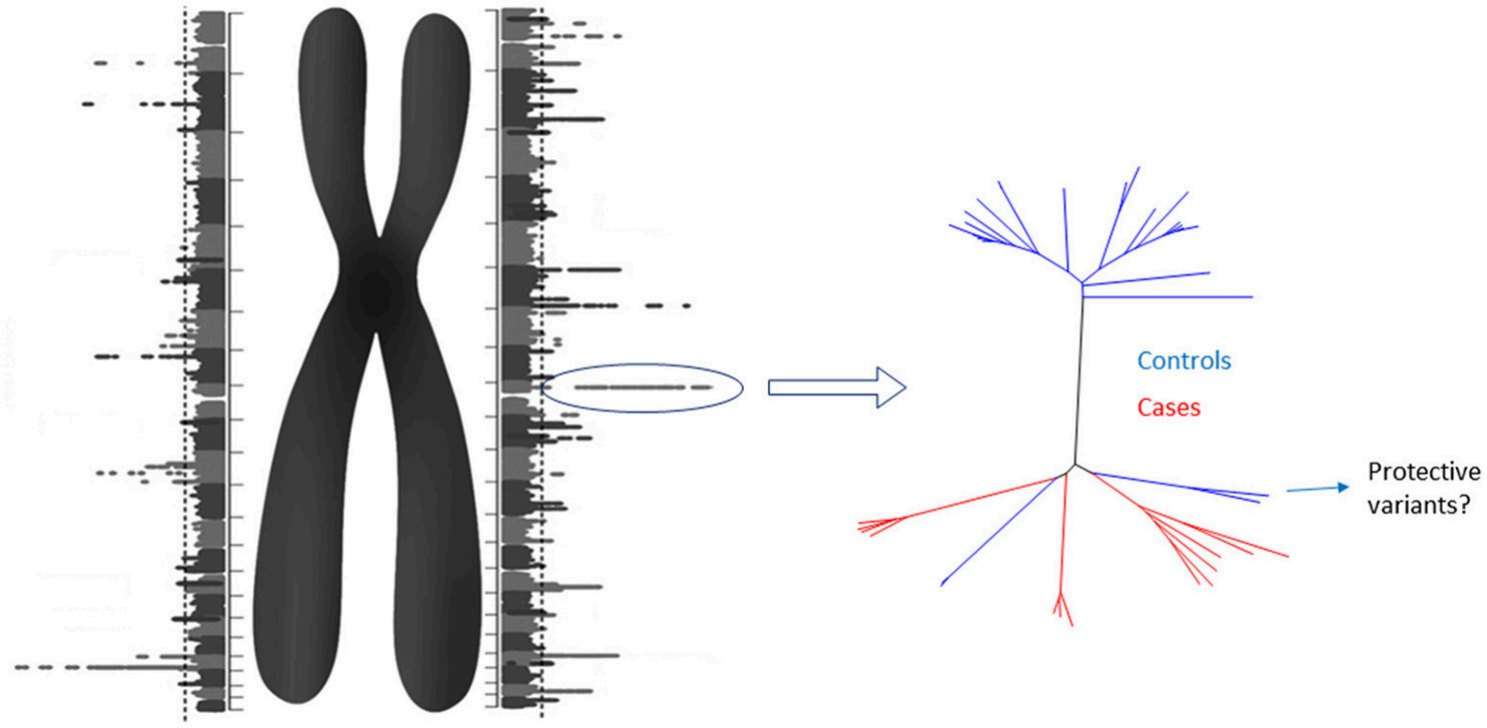
The Diploid Manhattan Plot: To achieve personalized medicine we need to isolate individual diploid genomes and compare them with a reference genome. Each homologous chromosome can be compared with the regular “haploid reference genome.” Thus, two GWAS that include the individual physical context of each genetic variant (including structural variants) will be produced. Since those DNA stretches would have a manageable size, the highest individual hits could be selected for further characterization comparing them with cases and control diploid genomes. For low penetrance diseases, protective SNPs in cis could be found. Diploid genomes have the potential to close the gap of the missing heritability problem.

Overall, to enable the rising field of personalized medicine, we need to unwind the whole genomic information in our diploid cells and elucidate what contributes to health and disease. The field of personalized medicine has to lead the change from “haploid” genomes to the real diploid ones since it is not only the wealthiest genomic area but also the one with a potential higher impact in the society. Therefore, it is paramount that we start a new genomic generation with a diploid revolution using the resources that have just been developed.

## Author contributions

The author confirms being the sole contributor of this work with the fruitful discussions mentioned in the acknowledgments.

### Conflict of interest statement

The author declares that the research was conducted in the absence of any commercial or financial relationships that could be construed as a potential conflict of interest.
